# Cognitive behavioural therapy in elderly type 2 diabetes patients with minor depression or mild major depression: study protocol of a randomized controlled trial (MIND-DIA)

**DOI:** 10.1186/1471-2318-10-21

**Published:** 2010-05-04

**Authors:** Frank Petrak, Martin Hautzinger, Kristin Plack, Kai Kronfeld, Christian Ruckes, Stephan Herpertz, Matthias J Müller

**Affiliations:** 1 Clinic of Psychosomatic Medicine and Psychotherapy, LWL-University Clinic Bochum, Ruhr-University Bochum, Bochum, Germany; 2 Department of Psychology, Clinical Psychology and Psychotherapy, Eberhard Karls University, Tübingen, Germany; 3 Department of Clinical Psychology and Psychotherapy, Johannes Gutenberg-University, Mainz, Germany; 4 Interdisciplinary Centre for Clinical Trials Mainz (IZKS Mainz), University Medical Centre, Johannes Gutenberg-University, Mainz, Germany; 5 Clinic for Psychiatry and Psychotherapy Giessen and Clinic for Psychiatry and Psychotherapy Marburg-Süd, Germany

## Abstract

**Background:**

The global prevalence of diabetes among adults will be 6.4% in 2010 and will increase to 7.7% by 2030. Diabetes doubles the odds of depression, and 9% of patients with diabetes are affected by depressive disorders. When subclinical depression is included, the proportion of patients who have clinically relevant depressive symptoms increases to 26%. In patients aged over 65 years, the interaction of diabetes and depression has predicted increased mortality, complications, disability, and earlier occurrence of all of these adverse outcomes. These deleterious effects were observed even in minor depression, where the risk of mortality within 7 years was 4.9 times higher compared with diabetes patients who did not have depressive symptoms. In this paper we describe the design and methods of the Minor Depression and Diabetes trial, a clinical trial within the 'Competence Network for Diabetes mellitus', which is funded by the German Federal Ministry of Education and Research.

**Methods/Design:**

Patients' inclusion criteria are: Type 2 diabetes mellitus, 65 to 85 years of age, 3 to 6 depressive symptoms (minor depression or mild major depression). Our aim is to compare the efficacy of diabetes-specific cognitive behavioural therapy adapted for the elderly vs. intensified treatment as usual vs. a guided self-help intervention regarding improvement of health related quality of life as the primary outcome. The trial will be conducted as a multicentre, open, observer-blinded, parallel group (3 groups) randomized controlled trial. Patients will be randomized to one of the three treatment conditions. After 12 weeks of open-label therapy in all treatment conditions, both group interventions will be reduced to one session per month during the one-year long-term phase of the trial. At the one-year follow-up, all groups will be re-examined regarding the primary and secondary parameters, for example reduction of depressive symptoms, prevention of moderate/severe major depression, improvement of glycaemic control, mortality, and cost effectiveness. Depending on additional funding, the sample will be continuously observed as a prospective cohort; the primary outcome will be changed to mortality for all subsequent follow-up measurements.

**Trial registration:**

Current Controlled Trials Register (ISRCTN58007098).

## Background

Diabetes is a worldwide problem with devastating human, social, and economic impacts. The global prevalence of diabetes among adults aged 20-79 years will be 6.4%, affecting 285 million adults, in 2010 and will increase to 7.7% (439 million adults) by 2030 [1]. The increase in the proportion of people 65 years of age and older appears to be an important demographic change that affects the prevalence of diabetes across the world [2]. Even when the known high rate of undiagnosed type 2 diabetes [3] is not taken into account, it can be estimated that more than eight million people are currently affected by diabetes in Germany [4]. With regard to the German population, the prevalence of diagnosed diabetes is nearly 8%; if we consider the estimated number of unreported cases, the prevalence actually is nearly 10% [4]. In addition to medical complications, e.g. microvascular complications [5], coronary heart disease [6], polyneuropathy [7], and vascular consequences [8], diabetes is extremely costly in economic terms. As a result of the high burden of illness and related comorbidities, diabetes has a considerable impact on quality of life, mortality, and the social insurance system. The direct medical expenditures as well as indirect costs attributable to diabetes in the US were estimated at $174 billion for 2007 [9], including $116 billion in excess medical care and $58 billion in reduced national productivity. In Germany, the total cost of diabetes was calculated at € 22 billion for the year 2001 [10]. Thus, the tremendous medical and economic burdens of diabetes make the disease an important clinical and public health problem.

There is an additional increase in health-service costs of 50%-75% when depression occurs together with diabetes [11]. People with diabetes are twice as likely to suffer from depression compared with the general population, with a pooled prevalence of 9% for depressive disorders based on diagnostic interviews and 26% based on self-report scales in controlled studies [12]. There is a strong body of evidence for multiple adverse interactions between diabetes and depression. Thus, depression is considered to be a risk factor for the development of diabetes mellitus. Moreover, the comorbidity of depression and diabetes is associated with adverse diabetes outcomes. Among patients aged over 65 years, the interaction of diabetes and depression has predicted increased mortality, complications, and disability, as well as an earlier occurrence of all these adverse outcomes. These deleterious effects were observed even in minor depression, where the risk of mortality within seven years was 4.9 times higher compared with diabetes patients who did not have depressive symptoms [13]. The elderly are the fastest growing segment of the European population. Depressive symptoms are a major public health problem in this population subgroup because they are common and are associated with considerable morbidity and increased mortality. Prevalence rates of 9.7% were shown in a German sample of primary-care patients aged 75 years and older [14]. However, most elderly persons who have clinically significant depressive symptoms do not meet the diagnostic criteria for major depression or dysthymic disorder; hence their depressive conditions are described as minor, subsyndromal, or subthreshold depression.

Depression has a strong impact not only on medical outcomes in diabetes but also on psychological and social outcomes [15,16]; therefore, the treatment of comorbid depression is increasingly considered essential for the clinical care of diabetes patients [17]. The described summary of the evidence for randomised controlled trials (RCT) treatments for depression in diabetes was based on a PubMed search. Eleven trials addressed the treatment of depressed diabetes patients in RCTs and were classified according to the studied interventions [18]. Counselling [19] and cognitive behavioural therapy (CBT) [20] demonstrated positive results in two trials; however, generalisability was restricted due to methodological limitations (small sample sizes, six-months follow-up, one trial site, and CBT not specific for diabetes). The most recent trial, which evaluated supportive psychotherapy, showed moderate improvement in depression according to post-treatment evaluation but not follow-up data [21]. Three RCTs [22-24] showed that antidepressants were superior to placebo in treating full-blown major depression. In the only RCT involving older, mildly depressed diabetes patients, paroxetine demonstrated no statistically significant differences compared with placebo therapy regarding health related quality of life (HRQoL), glycaemic control, or symptoms of depression after six months [25]. The effectiveness of algorithm-based, flexible interventions using a combination of psychological and pharmacological treatments compared with standard care was evaluated in four RCTs. The psychological modules of these treatments included problem-solving training [26,27], counselling [28], and interpersonal therapy [29]. Antidepressants were given according to the patients' preferences or following a predefined treatment algorithm. Three trials [25-27] provided evidence of successful treatment of depression. An additional RCT, which included elderly depressed diabetes patients (among others), showed that patients in the mixed treatment condition were less likely to die over the course of a five year interval than patients treated with usual care (adjusted hazard ratio 0.49, 95% CI [0.24, 0.98]) [28]. The mixed approaches provide at present the best available scientific evidence of successful treatment of depression.

In sum, there are encouraging findings that depression can be treated successfully in diabetes patients. Regarding metabolic control, SSRIs may have moderately beneficial results in trials with sufficient statistical power, while CBT and counselling for depression seem to have relatively positive short-term results. However, the superiority of one of these two approaches has not been demonstrated yet; neither has it been possible to generalise these results.

Up to now, no trial was found that directly compared cognitive behavioural therapy (CBT) with intensified treatment as usual (TAU) for older patients with diabetes type 2 and minor depression or mild major depression. Therefore, the aim of the present trial is to evaluate the efficacy of diabetes-specific CBT vs. an intensified TAU vs. a guided self-help intervention (SH) among elderly patients (65-85 years of age). CBT treatment has been tested in several trials and has been approved for treating depression among the elderly. TAU is an intensified form of the standard procedure of the German health care and health insurance systems. Currently, the best treatment for minor or mild depression among elderly diabetes patients has not been identified. Antidepressants are not superior to placebo, and psychosocial interventions have not been tested in this patient group. Hence, TAU is the best control group (with reasonable safety aspects) to test the superiority of a new psychosocial treatment. In order to control for the effect of a group intervention and to demonstrate specific efficacy, the guided self-help intervention 'Successful ageing with Diabetes' (SH) is a reasonable comparator and represents a usual group setting in Germany.

## Methods/Design

### Trial objectives and endpoints

The primary hypothesis of the MIND-DIA trial is that CBT is significantly and clinically more effective than TAU in terms of improvement of health related quality of life (HRQoL) at a one-year follow-up. Therefore, HRQoL (mental component) is analysed as the primary outcome parameter of the present trial.

The secondary hypotheses comprise the assumptions that CBT offers a specific advantage and is significantly more effective than SH, as demonstrated by a significantly greater improvement in HRQoL compared with SH groups at the one-year follow-up. Furthermore, it is surmised that at the one-year follow-up CBT will be found to be significantly more effective than TAU in terms of reduction of depressive symptoms and prevention of moderate/severe major depression, improvement of glycaemic control (HbA1c), prevention of mortality (yearly follow-up evaluations with mortality as an additional endpoint are planned depending on subsequent funding), and cost effectiveness (direct treatment costs: medication, hospitalisation and other costs over a period of one year).

### Trial design

**Figure 1:**
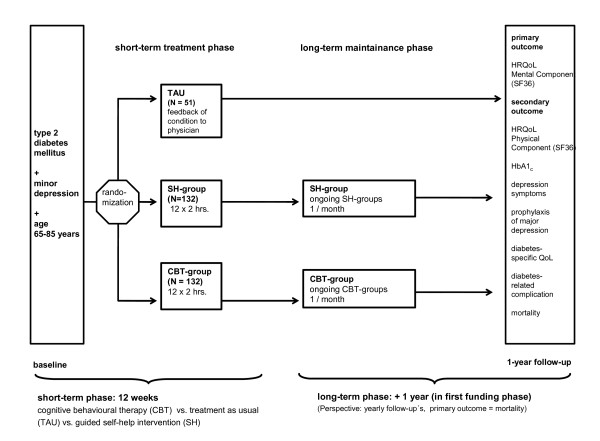
**Design of the MIND-DIA trial**.

The trial will be conducted as a multicentre, open, observer-blinded, parallel groups (3 groups) randomized controlled trial (RCT). 'Observer-blinded' means that treatment evaluations will be conducted by blinded assessors. Further blinding is not possible, given the comparison of clearly identifiable group interventions with treatment as usual. All eligible type 2 diabetes patients within the age range of 65 to 85 years who give informed consent will be screened at the participating trial sites in the multicentre trial. All patients with minor depression or mild major depression (3 to 6 depression symptoms according to DSM-IV-TR criteria) will be included in the trial, provided that they meet the other inclusion and exclusion criteria. Patients included in the MIND-DIA trial will be randomized to one of the three treatment conditions: CBT, SH or TAU. In all groups patients will receive diabetological treatment as usual by their treating physicians during the trial. After 12 weeks of open-label therapy, both group interventions will be reduced to one session per month in the long-term phase of the trial (12 additional months). At the 12-month follow-up, all groups will be re-examined regarding the primary outcome variable and all secondary variables. A first analysis is planned after the end of the short-term phase of the RCT without the analyses of the parameters for depression and HRQoL. Therefore, this interim analysis will not influence the primary results or the conduct of the trial. For the first funding phase, the duration of the MIND-DIA trial is expected to be approximately 36 months. Recruitment of patients started in May 2009. The trial design is shown in figure 1.

### Trial sample

A total of 315 subjects will be included in the trial. Patients will be recruited in approximately 20 centres that specialise in diabetes treatment in the Rhine-Main and Ruhr areas. In the participating centres, all 65- to 85-year-old patients with type 2 diabetes who give informed consent will be screened in a two-stage procedure (Patient Health Questionnaire (PHQ-9) [30] followed by the Structured Clinical Interview for DSM IV Disorders (SCID) [31]. All patients with minor depression or mild major depression will be included in the trial. Other inclusion and exclusion criteria of the trial are listed below.

### Inclusion criteria

• type 2 diabetes mellitus diagnosed at least 6 months before entering the trial

• 65 to 85 years of age

• minor depression (adapted from the DSM-IV-TR research criteria: we require 3 to 4 symptoms rather than 2 to 4 symptoms or mild major depression (according to DSM-IV-TR criteria, 5 to 6 depressive symptoms); a past history of major depression is not an exclusion criterion

• living near the coordinating institution where treatment will take place

• written informed consent given

### Exclusion criteria

• serious violent, homicidal, or suicidal ideation, particularly clinically significant suicide risk or history of attempted suicide within the past 2 years

• history of schizophrenia, psychotic symptoms, or bipolar disorder

• organic brain syndrome, dementia, or substantial cognitive impairment (according to the three-step MCI screening)

• alcohol or substance abuse or dependence in the past 12 months (other than nicotine abuse/dependence)

• insufficient ability to understand German

• regular participation in a self-help group (minimum 4 sessions in the past 12 months)

• psychotherapy (in the past 3 months)

• bereavement and grief reaction (loss < 3 months)

• planned hospital admission within the next 3 months

• medical contraindication for physical activity

• history of severe, acute or chronic medical disorder (other than diabetes), which probably would impede commitment to the trial or lead to biased trial results (based on the critical appraisal of the investigator)

• patients who have taken antidepressants or mood-stabilising medication regularly over the 30 days prior to screening, patients who have taken fluoxetine regularly over the 60 days prior to screening, or patients on depot neuroleptic medication over the 5 inter-injection intervals prior to screening

• blood chemistry ALT and/or AST value(s) greater than or equal to three times the upper limit of normal prior to randomization; estimated GFR lower than 30; blood chemistry TSH values lower than 0.5 mlU/L or greater than 5 mlU/L adapted to reference ranges of the laboratory

• legal incompetence or legal guardianship

• participation in competing trials

### Trial interventions

Patients included in the MIND-DIA trial will be randomized to one of the three treatment arms. Within group interventions, all sessions will be videotaped and supervised by psychotherapists. Adherence to treatment will be evaluated by a rating system.

(1) CBT is a manualised diabetes-specific cognitive behavioural therapy, delivered by trained psychologists in small groups (4 to 8 participants) in an outpatient setting. CBT is based on a manualised programme (12 weekly sessions) designed for older adults with type 2 diabetes; the treatment includes cognitive and behavioural strategies to overcome depression and diminish diabetes-related distress, reduce perceived barriers to various aspects of self-management, increase physical activity, and enhance coping skills. Central elements are psychoeducation, support, problem solving, pleasant activities, scheduling of physical activity by use of pedometers, thought-control techniques, cognitive restructuring, Socratic dialogue, training in social skills and interpersonal contact, crisis intervention, and emergency planning. In terms of a workbook, theoretical background, worksheets, and exercises are given to the patients. An important element of the CBT condition is the adoption of pedometers to increase patients' activity levels. For seven days prior to the start of the intervention, patients will wear a sealed pedometer to determine their baseline activity. During the first week of the trial, patients will not change their usual activity level. At the start of the intervention, there will be one session in which information will be provided to the patients about the link between the amount of walking and improvements in health and about general consequences of doing more walking. Patients will be asked to set themselves specific weekly (or monthly) goals regarding the number of steps they will take based on their individual baseline measurements. In addition to the daily step measurements, the number of steps will be recorded by the pedometer on a weekly basis. Furthermore, patients will receive weekly feedback on their physical activity level in the form of graphic and written information. After 12 weeks of open-label therapy, the CBT intervention will be reduced to one session per month during the long-term phase of the trial. The topics of the short-term sessions will be replicated during the long-term phase in order to increase the therapeutic power of the intervention.

(2) The manualised, guided self-help intervention 'Successful ageing with Diabetes' (SH), with a focus on living and ageing with diabetes, will be delivered by trained moderators (elder-care nurses, nurses, educators, or others). Moderators should pay attention, be empathetic, and give support to all patients. The guided self-help intervention will provide an intact community and a sense of belonging. This procedure will enable the investigators to control for unspecific group effects due to participation in a social network of people who share the same problems; such participation may reduce social or emotional isolation. The moderators should promote reciprocal caring and the sharing of relevant information regarding diabetes and ageing. The primary value of this group condition will be the mutual aid offered by members to one another. Therefore, no formal therapeutic aspects will be involved; moderators should restrict their function to guiding the group and giving support. The intervention is based on a manualised programme, which comprises only recommendations for group sessions. Patients primarily choose the topics or group activities. Thematically, the intervention modules will be comparable to the programmes of German self-help groups; such programmes consist of spontaneous conversation or several group activities.

(3) The participants in the TAU group and their treating physicians will receive diagnostic feedback regarding minor or mild depressive symptoms and cognitive function. Furthermore, feedback on the therapeutic options will be given to the physicians if minor or mild depressive symptoms are diagnosed. For minor depression or depressive symptoms on a subclinical level, the following treatment options, including combinations of them, are generally possible:

• 'watchful waiting' (to await the spontaneous remission of the disorder)

• psychotherapy and/or physical activity

• currently, there is no scientific evidence for medical treatment with antidepressants in elderly diabetes patients with minor depression, but a treatment attempt with a well-tolerated antidepressant (e.g. sertraline or citalopram) could be an optional medical intervention.

However, any treatment option may be chosen, since care as usual for sub-threshold depression is currently not formalised.

### Outcome measures

The primary outcome variable is HRQoL as measured by the Short Form-36 [32]: z-values will be obtained by z-transformation of the SF-36 Mental Component Summary score based on means and standard deviations of age- and gender-matched reference groups from the German general population. This transformation will be performed because it is known that in some respects HRQoL decreases differently with age for men and women. Because the level of depressive symptoms is already relatively low in patients with subthreshold depression compared with patients who have major depression (notwithstanding a comparably low HRQoL), the possibility for improvement is reduced ('bottom effect'). Hence, when one assesses the efficacy of an intervention in this specific group, it seems reasonable to analyse, in additional studies, other important variables, e.g. HRQoL, as the primary outcome variable and depressive symptoms as a secondary variable among others. Results of our own research demonstrated that HRQoL is extremely reduced among diabetes patients with depressive symptoms: Differences in SF-36 z-values were -1.4 for the physical and -1.9 for the mental component, a finding that indicates that a bottom effect is not to be expected in HRQoL.

Secondary outcome variables are: HRQoL (physical component as measured by the Physical Component Summary Score), reduction of depressive symptoms (Quick Inventory of Depressive Symptomatology-Clinician Rated, QIDS-C-16 [33]; Hamilton Depression Scale, HAMD [34]), prevention of moderate/severe major depression (Depression module, SCID), improvement of glycaemic control (HbA1_c_), prevention of mortality, and cost-effectiveness. Standardised rater training of evaluators who were blind to treatment conditions was carried out for observer-derived depression assessments (QIDS-C, HAMD, SCID).

Other psychometric questionnaires used in the present trial are: PAID, Problem Areas in Diabetes [35,36]; FKV-15, Freiburger Fragebogen zur Krankheitsverarbeitung (15-Item-Version) [37]; RSE, Rosenberg Self-Esteem Scale [38]; SDSCA, Summary of diabetes self-care activities measure [39]; H-Skala, Hopelessness Scale [40]; and K-INK, Inkongruenzfragebogen [41]. Economic evaluation will be performed alongside the MIND-DIA clinical trial over the course of the complete trial period. Costs and effects will be discounted at the rate recommended by the German guidelines for economic evaluation issued by the Institute for Quality and Efficiency in the Health Care Sector (IQWiG); the rate currently is 3%. The three outcomes estimated for the economic analysis will be depression-free years, quality adjusted life years (QALYs), and cumulative cost accrued in each arm of the trial. A detailed protocol of the cost-effectiveness analysis alongside the MIND-DIA study was published previously [42].

In addition, mild cognitive impairment (MCI) will be identified by a three-step MCI diagnostic instrument. The MCI parameter will be used as a predictor for treatment response in the CBT group. It is expected that patients with MCI will show poorer responses to the treatment compared with patients without MCI. In a first step all patients will be screened by the semi-quantitative interview according to Strawbridge [43] via telephone or face to face by research assistants. Those patients who report memory decline within the past year will be screened in a second step by the Mini-Mental State Examination [44]. Patients who score less than 25 points but more than 16 points on the MMSE will be screened in a third step by the following instruments: Clinical Dementia Rating (CDR) [45], Syndrom Kurztest (SKT) [46], and Mehrfachwahl-Wortschatz-Intelligenztest (MWTB) [47]. Early Alzheimer's dementia or substantial cognitive impairment will be an exclusion criterion for the trial. All patients who are excluded in the baseline phase of the trial will receive support to arrange an elaborate medical diagnostic evaluation. Initially, MCI-positive patients will be included in the trial but screened again at a 1-year follow-up by the MMSE. Endpoints, measuring instruments, and time of data collection are shown in table 1.

**Table 1:** Endpoints, measuring instruments, and time of data collection

Endpoint of the economic evaluation	Derived from/questionnaire name	Time of measurement (Month)					
		
		Baseline	M 3	M 6	M 9	M 12	M 15
Incidence rate of depression	Depression module of the SCID	**X**	**X**				**X**

Severity of depression	QIDS/Hamilton Scale	**X**	**X**			**X**	**X**

QALYs	SF-36 EQ-5D	**X**	**X**	**X**	**X**	**X**	**X**

MCI (three-step screening)	Interview of Strawbridge MMSE CDR SKT + MWTB	**X**					X*

Health sector and patients' costs	Cost questionnaire	**X**		**X**		**X**	**X**

Laboratory parameters	Safety parameters: ALT, AST, creatinine, TSH	**X**					
	HbA1c	**X**	**X**	**X**	**X**	**X**	**X**

Diabetes-related emotional distress, coping, diabetes self-care activities	PAID SDSCA FKV-15	**X**	**X**			**X**	**X**

Interpersonal aspects	SE H-Skala K-INK	**X**	**X**			**X**	**X**

*those patients with a positive MCI screening at baseline

### Statistical analysis

#### Sample size calculation

The power calculation is based on expected differences in SF-36 z-scores. Based on our own comparison of type 2 diabetes patients with mainly untreated depression versus non-depressed patients, we assume differences of δ = 0.6 between CBT and TAU and of δ = 0.4 between CBT and SH. For the latter comparison, a significant difference can be detected with a power of 90% if n = 132 patients per intervention group are enrolled (2-sided t-test, α = 0.05). Given 132 patients in the CBT group, it is sufficient to enroll 51 patients in the TAU group to achieve a power of 95% for the comparison CBT vs. TAU. We therefore plan to recruit a total number of 315 patients (132 in CBT, 132 in SH, and 51 in TAU). We expect a rate of loss to follow-up of 20% in light of our experiences in the ongoing Depression and Diabetes (DAD) study and assuming that mortality will play a role in our sample because of the age of the patients. Analyses will be performed using an intent-to-treat principle (ITT) that includes all randomised subjects in the groups to which they were assigned. The last observation carried forward method (LOCF) will be used for the handling of missing data due to dropouts. The primary population for analysis is the ITT population. To fully appreciate the potential influences of missing responses and the LOCF method chosen as imputation procedure, additional sensitivity analyses examining the effects of different imputation methods will be performed and discussed.

#### Analysis of outcome variables

There will be one analysis of depression and health related quality of life after completion of the trial, i.e. no interim analyses are planned for the primary parameters. The primary response variable is the z-transformed SF-36 Mental Component Summary (MCS) score at the one-year follow-up. We plan to employ a linear regression model of z-transformed SF-36 score on type of intervention and baseline score. Additional variables (e.g. gender, age, centre, and illness severity) may be included in the model, if indicated.

The hypotheses will be ordered hierarchically (1: CBT vs. TAU, 2: CBT vs. SH); the second hypothesis will be tested at a two-sided 5% level only if the first test is significant at a two-sided 5% level. Analyses will be performed using an intent-to-treat analysis, and the last observation carried forward method will be used for the handling of missing data. Secondary outcomes (HRQoL (physical component), severity of depression, prophylaxis of moderate/severe major depression, glycaemic control (HbA1c), and mortality) will be analysed primarily in a descriptive manner. For binary response variables, the numbers and proportion of responders will be presented together with 95% confidence intervals for the proportions; continuous variables will be analysed by use of summary statistics. If differences between intervention groups are investigated, outcomes will be adjusted for baseline values through use of appropriate models (linear, logistic, or Poisson regression); however, the p-values for such secondary analyses may not be interpreted as confirmatory findings. Subgroup analyses stratified for gender (male/female), mild cognitive impairment (yes/no), and number of previous depressive episodes (<3, ≥3) also will be carried out. These analyses will be conducted using the same methods as those for the full population. Time to onset of moderate/severe major depression and conversion rates to dementia will be analysed using Kaplan-Maier estimates.

#### Safety

Differences between the treatment groups regarding rates of mortality and suicidality will be described descriptively. P-values from Fisher's exact test for pairwise comparison may be calculated but not interpreted in a confirmatory manner.

#### Ethical aspects

The procedures set out in the trial protocol regarding the conduct, evaluation, and documentation of this trial are designed to ensure that all persons involved in the trial abide by Good Clinical Practice (GCP) and the ethical principles described in the current revision of the Declaration of Helsinki. Clinical monitoring, data management, and biometry according to GCP will be conducted by IZKS Mainz. The trial will be carried out in keeping with local legal and regulatory requirements. All patients will be in treatment, regularly seen by diabetologists and trial clinicians. Before being admitted to the clinical trial, the patients must agree to participate after the nature, scope, and possibleconsequences of the trial have been explained in a form understandable to them. The patients must give written informed consent. A copy of the signed consent document must be given to each patient. The documents must be in language that is understandable to the patients and must specify who informed them. During the trial, patients will be identified solely by means of year of birth and an individual identification code (patient number, randomisation number). For protection of these data, organisational procedures will be implemented to prevent distribution of the data to unauthorised persons. The appropriate regulations of local data legislation will be followed in their entirety. Five independent ethics committees voted their approval of these procedures. The main ethics committee for the MIND-DIA trial is the local Medical Ethics Committee (Ethikkommission bei der Landesärztekammer Hessen); approval has been given on 09/04/2009. Further ethic committees which approved the MIND-DIA trail are: the local Medical Ethics Committee (Ethikkommission der Ärztekammer Nordrhein), the local Medical Ethics Committee (Ethikkommission der Ärztekammer Westfalen-Lippe) and the Ethics Committees of the Ruhr-University Bochum and the Johann-Wolfgang Goethe University, Frankfurt.

## Competing interests

The authors declare that they have no competing interests.

## Authors' contributions

FP coordinator and principal investigator (PI), MJM coordinating investigator, MH applicant and investigator, KK clinical trial specialist, CR biometrician. All authors developed the design and methods for the clinical trial. KP and FP wrote the manuscript. CR provided support relating to the statistical analysis. All co-authors read, edited, and approved the final manuscript. All authors participated in the work sufficiently to take public responsibility for their respective parts of the paper.

## Pre-publication history

The pre-publication history for this paper can be accessed here:

http://www.biomedcentral.com/1471-2318/10/21/prepub
